# The Effectiveness and Mechanism of *Toona sinensis* Extract Inhibit Attachment of Pandemic Influenza A (H1N1) Virus

**DOI:** 10.1155/2013/479718

**Published:** 2013-09-02

**Authors:** Huey-Ling You, Chung-Jen Chen, Hock-Liew Eng, Pei-Lin Liao, Sheng-Teng Huang

**Affiliations:** ^1^Department of Laboratory Medicine, Kaohsiung Chang Gung Memorial Hospital and Chang Gung University College of Medicine, Kaohsiung 83301, Taiwan; ^2^Division of Rheumatology, Allergy and Immunology, Kaohsiung Chang Gung Memorial Hospital and Chang Gung University College of Medicine, Kaohsiung 83301, Taiwan; ^3^Department of Pathology, Kaohsiung Chang Gung Memorial Hospital and Chang Gung University College of Medicine, Kaohsiung 83301, Taiwan; ^4^Department of Chinese Medicine and Mitochondrial Research Unit, Kaohsiung Chang Gung Memorial Hospital and Chang Gung University College of Medicine, Kaohsiung 83301, Taiwan; ^5^Department of Chinese Medicine, Chang Gung Memorial Hospital, Kaohsiung Medical Center, Chang Gung University College of Medicine, Taiwan

## Abstract

TSL-1 is a fraction of the aqueous extract from the tender leaf of *Toona sinensis Roem*, a nutritious vegetable. The pandemic influenza A (H1N1) virus is a recently described, rapidly contagious respiratory pathogen which can cause acute respiratory distress syndrome (ARDS) and poses a major public health threat. In this study, we found that TSL-1 inhibited viral yields on MDCK plaque formation by pandemic influenza A (H1N1) virus on infected A549 cells with high selectivity index. Meanwhile, TSL-1 also suppressed viral genome loads in infected A549 cells, quantified by qRT-PCR. This study further demonstrated that TSL-1 inhibited pandemic influenza A (H1N1) virus activity through preventing attachment of A549 cells but not penetration. TSL-1 inhibited viral attachment through significant downregulation of adhesion molecules and chemokines (VCAM-1, ICAM-1, E-selectin, IL-8, and fractalkine) compared to Amantadine. Our results suggest that TSL-1 may be used as an alternative treatment and prophylaxis against pandemic influenza A (H1N1) virus.

## 1. Introduction

The 2009 pandemic influenza A (H1N1) virus, a new strain of virus identified in Mexico in April 2009, spread rapidly across the globe [1]. According to WHO statistics, this pandemic virus has killed more than 18,000 people around the world since 2009 [2]. In the past few years, there has been a worldwide effort to virus isolation, whole genome sequencing, and hemagglutination inhibition assay to confirm influenza A viruses, which has led to the depositing of more than 46,000 sequences in the Influenza Virus Resource of the National Center for Biotechnology Information (NCBI). Genomic analysis of the 2009 influenza A (H1N1) virus in humans indicates that it is closely related to common reassortant swine influenza A viruses isolated in North America, Europe, and Asia [3]. The 2009 pandemic influenza A (H1N1) virus is comprised of RNA segments from both North American and Eurasian swine influenza and from avian influenza viruses [3]. Morlighem et al. found that the 2009 pandemic influenza A (H1N1) virus had a high evolutionary rate [4]. Soon thereafter, oseltamivir-resistant 2009 pandemic influenza A (H1N1) virus was found [5]. It is therefore prudent to find new antiviral agents.

The tender leaves of *Toona sinensis* (TS) have been traditionally regarded as a nutritional supplement or vegetable in both Mainland China and Taiwan. In a recent report [6], there were no significant toxic effects on the biochemical and histopathological parameters of female mice treated with the extract of TS. The extracts from TS leaves have a wide range of biologic functions, including glycemic control, anti-LDL glycative activity [7], and improving cognitive function [8]. TS extracts have also been demonstrated to inhibit cancer cell growth in human ovarian cancer, human premyelocytic leukemia, human lung adenocarcinoma, and prostate cancer [9–13]. The mechanism of anticancer activity by TS could be due to the generation of reactive oxygen species and mitochondria-mediated apoptosis [11, 14]. Moreover, TS possesses antiangiogenic activity through suppressing VEGF-induced expression of metalloproteinase- (MMP-) 9 and MMP-2 on endothelial cells [15].

Traditional Chinese medicine (TCM) may be a great treasure for finding new antiviral agents against the influenza A (H1N1) virus. Wang et al. found that maxingshigan-yinqiaosan may be used as an alternative treatment for H1N1 influenza virus infection [16]. Hsieh et al. reported that maxingshigan tang abolished the entry of H1N1 influenza virus through regulation of PI3 K/AKT pathway [17]. The aqueous extracts of dandelion possess inhibited H1N1 replication in MDCK and A549 cells [18]. Although many TCM herbs have been found to have anti-influenza property, their mechanisms have not yet been clearly verified. In our previous study, we found that a major fraction from the tender leaves of TS, known as TSL-1, had a significant inhibitory effect on coronavirus, the family of virus that included SARS coronavirus which induced severe acute respiratory syndrome (SARS) with global outbreak in 2003 [19]. In addition, tender leaves of TS appear to be beneficial in preventing upper respiratory infection in our clinical experience.

As we know, the adhesion molecules are upregulated on alveolar epithelial cells in response to TNF-*α* stimulation or during bacterial or viral infection. We are the first to discover that the extract of *Toona sinensis* has anti-influenza effect through modulating adhesion molecules and chemokines. Herein, we analyzed whether TSL-1 has anti-influenza activity in cell culture and attempted to delineate the relevant mechanisms in our study. We concluded that TSL-1 may inhibit influenza virus infection and potentially be a promising treatment to protect the host against pandemic influenza A (H1N1) virus.

## 2. Materials and Methods

### 2.1. Preparation and Fractionation of Extracts from *Toona sinensis*

TSL-1 is a fraction of crude extract from the tender leaf of *Toona sinensis Roem* prepared according to previous study [12]. In brief, 100 g of tender leaves yields approximately 5-6 g of lyophilized TSL-1 powder. This aqueous extract of TS was characterized by high-performance liquid chromatography (HPLC). Nine compounds (gallic acid, methyl gallate, ethyl gallate, kaempferol, kaempferol 3-O-*β*-D-glucoside, quercetin, quercitrin, quercetin 3-O-*β*-D-glucoside, and rutin) were identified from TS as previously described [13]. In the antiviral assay against pandemic influenza A (H1N1) virus, they were dissolved in water and then diluted in cell culture medium.

### 2.2. Virus Preparation and Cell Culture

The pandemic influenza A (H1N1) virus (A/California/07/2009) was isolated from the 2009 outbreak and supplied by the clinical virology laboratory of Kaohsiung Chang Gung Memorial Hospital, Taiwan. The human lung carcinoma (A549) cells and Madin-Darby canine kidney (MDCK) cells were purchased from ATCC (Manassas, VA, USA). Both cell lines were grown at 37°C in MEM medium (Invitrogen Life Technologies, Carlsbad, CA, USA) supplemented with 10% heated-inactivated FBS, 250 ng/mL amphotericin B, and 100 *μ*g/mL penicillin and streptomycin. Influenza A virus strain was prepared by infecting MDCK cell cultures. Supernatants from infected cultures were collected 3 days after infection, and aliquots were stored at −80°C until use. Virus titer was determined by cytopathic effect (CPE) and expressed as TCID50 (50% tissue culture infective dose).

### 2.3. Cytotoxicity Assay and Selectivity Index (SI)

The cytotoxic effect of TSL-1 on growing A549 and MDCK cells was determined by XTT (tetrazolium hydroxide salt) assay according to the manufacturer's instructions (Roche Molecular Diagnostics, Germany). Briefly, cells (1 × 10^5^ cells/well) were plated into 96-well culture plates for an incubation period of 24 hours. Then, various concentrations of TSL-1 (0–300 *μ*g/mL) were supplemented immediately. After incubation at 35°C with 5% CO_2_ for 3 days, XTT reagent was added and incubated for 4 hours. The absorbance of the resulting solution was measured spectrophotometrically at A570 nm (Sunrise, TECAN) with a reference of A650 nm. Each experiment was carried out in triplicate and performed at least thrice separately. The 50% cytotoxic concentration (CC_50_) of TSL-1 was calculated. The selectivity index was calculated as the ratio of CC_50_ : EC_50_.

### 2.4. Virus Infection

Monolayer of A549 cells at a concentration of 3 × 10^5^ cells/mL was infected with the pandemic influenza A (H1N1) virus (3 × 10^5^ PFU/mL). After 1 hour, the solution was removed; the cells were washed twice with phosphate buffer saline (PBS) and supplemented with growth media contained TSL-1 at different concentrations. Amantadine was used as a positive control. Cells were harvested at 72 hours after-infection, and the viral yield was estimated by quantitative RT-PCR and plaque assay on MDCK cells. As a vehicle control, the infected cells incubated in TSL-1-free medium were included throughout the experiment.

### 2.5. Plaque Assay

Confluent monolayer of A549 cells in 6-well plates was infected with pandemic influenza A (H1N1) virus (3 × 10^5^ PFU/mL) for 1 hour and then replaced with medium containing TSL-1 of various concentrations. Viruses were harvested at 72 hours after infection using a process through three cycles of freezing and thawing and clarified by low speed centrifugation (500 g for 10 min). Virus yields in the culture supernatants were assayed by the plaque assay in MDCK cells. Briefly, confluent monolayer MDCK cells cultured in a 6-well tissue culture plate (2 × 10^5^ cells/cm^2^) were infected with the supernatants. After 60 minutes for virus adsorption, the solution was removed and the cells were washed twice with prewarmed MEM medium and replaced with overlay medium (MEM containing 2 *μ*g/mL trypsin, 5% low melting agarose, without serum), containing Amantadine or TSL-1 at different concentrations. After incubating cultures for 72 hours at 35°C with 5% CO_2_, monolayer was fixed with 4% formaldehyde solution for 60 minutes. The agarose was then removed by flowing water and stained with 1% (w/v) crystal violet solution. Assays were performed in 6-well plates in triplicate. The plaques were counted by Quantity One version 4.6.5 (Bio-Rad Laboratories) examination, and percentage of plaque inhibition was calculated as relative to the vehicle control. A required concentration to reduce the 50% plaque number (EC_50_) was calculated by regression analysis of the dose-response curves generated from these data.

### 2.6. Quantification of Pandemic Influenza A (H1N1) Virus

Total nucleic acids were extracted on a MagNaPure instrument (Roche Molecular Diagnostics, Germany) using the manufacturer's external lysis protocol and extraction reagents (Total Nucleic Acid Isolation Kit; Roche Molecular Diagnostics, Germany) to yield 100 *μ*L of eluate. All PCR assays were performed using standard precaution to avoid contamination. The primers (sense) 5-AAG ACC AAT CCT GTC ACCTCT GA-3 and (antisense) 5-CAA AGC GTC TAC GCTGCA GTC C-3 amplify a 104-base pair fragment in the M1 gene of influenza A. The influenza A specific probe: FAM (6-carboxyfluorescein)-5-TTT GTG TTC ACG CTC ACC GT-3-TAMRA (6-carboxytetramethylrhodamine) [20]. For the quantitative assay, each 20 *μ*L RT-PCR mixture contained 1x Universal PCR Master Mix, 1x MultiScribe, and RNAse Inhibitor, 250 nM each of forward and reverse primer, 100 nM of TaqMan probe, and 5 *μ*L of extracted RNA, or water for no template controls. The reactions were performed and analyzed in a 7500 sequence Detection System (PRISM, Applied Biosystems, USA) under the following conditions: 30 minutes at 48°C and 10 minutes at 95°C, followed by 40 cycles of 30 seconds at 95°C and 1 minute at 60°C. Plasmids containing a known copy number of amplification targets were included in the real-time PCR assay to generate a standard curve for quantification of test samples.

### 2.7. Attachment Assay

The cells were seeded and incubated for 24 hours and prechilled at 4°C for 1 hour, and the mediums were aspirated. Then, the cells were infected with pandemic influenza A (H1N1) virus of 3 × 10^5^ PFU/mL and different indicated concentrations of TSL-1. After incubation at 4°C for another 3 hours, the mediums were aspirated to remove unabsorbed virus. The cells were washed with PBS three times and incubated for an additional 72 hours and assayed with quantitative RT-PCR (qRT-PCR) as described above.

### 2.8. Penetration Assay

The cells were grown in 24-well culture plates and prechilled at 4°C for 1 hour. The cells were then infected with pandemic influenza A (H1N1) virus of 3 × 10^5^ PFU/mL and incubated at 4°C for another 3 hours to allow the viral attachment to the cell surfaces. Then different concentrations of TSL-1 were supplemented to the cells and incubated at 37°C under 5% CO_2_ for 1 hour to maximize the penetration of viruses, after which the infected cells were treated with PBS (pH 11) for 1 minute to inactivate unpenetrating viruses. The PBS (pH 3) was supplemented immediately to neutralize alkaline PBS (pH 11). The neutral PBS was removed, and the cells were supplemented with fresh culture mediums. After incubation at 35°C for an additional 72 hours, the cells were assayed with qRT-PCR as described above.

### 2.9. Hemagglutination (HA) Inhibition Test

To follow the treatment procedures of attachment assay or penetration assay, the cell culture supernatant was collected. One hundred microliter of the supernatant with further fresh preparation 0.5% guinea pig erythrocytes suspension was added into the U-shaped 96-well microtitre plates and mixed thoroughly. The plate was incubated for 3 hours at 4°C. The RBC agglutination was observed. The experiments were repeated three times independently.

### 2.10. Neuraminidase (NA) Assay

To follow the treatment procedures of attachment assay or penetration assay, the cells were probed with neuraminidase antibody (Abcam, Cambridge, MA, USA) at 37°C for 3 hours. After being washed three times with PBS, the cells were incubated with horseradish peroxidase conjugated secondary antibody (Millipore, Bedford, MA, USA) for 2 hours at room temperature. The TMB solution (Cell signaling, Boston, MA, USA) was added and incubated for 5 minutes. After being added with stop solution (cell signaling), the absorbance of the resulting solution was measured spectrophotometrically at A450 nm (Sunrise, TECAN). The experiments were repeated three times independently.

### 2.11. ELISA for Sialic Acid

To follow the treatment procedures of attachment assay or penetration assay, the cell culture supernatant was collected. A human sialic acid-specific (SIGMA) was used to determine the levels of sialic acid in conditioned media collected from A549 cells. The experimental steps were carried out as described in the protocol provided by manufacturer. They were performed three times and each time was analyzed in duplicate, respectively.

### 2.12. ELISA for RANTES

Monolayer of A549 cells at a concentration of 3 × 10^5^ cells/mL was infected with the pandemic influenza A (H1N1) virus (3 × 10^5^ PFU/mL). After 1 hour, the solution was removed; the cells were washed twice with phosphate buffer saline (PBS) and supplemented with growth media contained TSL-1 at different concentrations. Cell supernatants were collected at 72 hours and as described in the protocol provided by manufacturer (R&D Systems, Inc., Minneapolis, MN, USA). They were performed three times and each time was analyzed in duplicate, respectively.

### 2.13. RNA Isolation and Quantitative Real-Time Polymerase Chain Reaction (qRT-PCR) Analysis

Total cellular RNA was isolated by lysis of cells in a guanidinium isothiocyanate buffer, followed by single step phenol-chloroform-isoamyl alcohol extraction procedure modified from that previously described [21]. Briefly, untreated or treated cells with TSL-1 were harvested and lysed in 4 M guanidinium isothiocyanate, 25 mM sodium citrate (pH 7.0), 0.5% sodium lauryl sarkosine and 0.1 M ß-mercaptoethanol. Sequentially, 1/10 volume of 2 M sodium acetate (pH 4.0), one volume of phenol, and 1/5 volume of chloroform-isoamyl alcohol (49 : 1, v : v) were added to the homogenate. After vigorous vortexing for 30 seconds, the solution was centrifuged at 10,000 ×g for 15 minutes at 4°C. After removal of the aqueous phase, RNA was precipitated by the addition of 0.5 mL isopropanol. The primers and probe were designed using Probefinder v2.45 (http://qpcr.probefinder.com/roche3.html). qRT-PCR primers and an appropriate probe were chosen by the UPL Assay Design Center. The free online software specifies a set of specific primers plus the TaqMan locked nucleic acid (LNA) probe from the Roche Universal ProbeLibrary collection. The primers and probe are shown in Table 1. Two hundred nanograms of total RNA from control, infected A549 cells and TSL-1 treatment infected A549 cells, were used for quantitative RT-PCR analysis. PCR was performed on the LightCycler Instrument (Roche Diagnostics, Penzberg, Germany). Reaction was performed using the RealTime ready RNA Virus Master kit (Roche Diagnostics, Penzberg, Germany) according to the manufacturer's instructions. The quantization of target genes was determined by the relative quantitative comparative threshold cycle (ΔΔCt) method using the GAPDH as the endogenous control, where Ct is the threshold cycle. The quantification for the target gene in test sample against reference samples (uninfected cells) was using the formula 2^−ΔΔCt^, where ΔΔCt = (Ct of target gene in each test sample − Ct of GAPDH in each test sample) − (average Ct of target gene in reference samples − average Ct of GAPDH in reference samples).

### 2.14. Western Blotting of Cell Lysates

Protein concentrations were determined by the Bradford method (Bio-Rad, CA, USA). Samples with equal amounts of proteins were subjected to 10% SDS-PAGE and transferred onto a polyvinylidene difluoride (PVDF) (Millipore, Bedford, MA, USA) membrane. The membrane was incubated at room temperature in blocking solution: 1% BSA (bovine serum albumin), 1% goat serum in PBS for 1 hour, followed by a 2-hour incubation in blocking solution containing an appropriate dilution (1 : 1000) of primary antibody, for example, anti-VCAM, anti-ICAM, anti-E-selectin, anti-fractalkine (CX3CL1), anti-IL-8, anti-Bcl-2 related protein (Bcl2-A1), anti-TLR3, and anti-tubulin antibody (NeoMarkers, Fremonk, CA, USA). After washing, the membrane was incubated in PBS containing goat anti-mouse IgG conjugated with horseradish peroxidase (Sigma, St. Louis, MO, USA) for 1 hour. The membrane was washed, and the positive signals were developed with chemiluminescence reagent (Amersham Pharmacia Biotech, Little Chalfont Buckinghamshire, England). Then the membrane was exposed to Fuji medical X-ray film (Fuji Ltd, Tokyo, Japan) for 3 minutes.

### 2.15. Statistical Analysis

All statistical analyses were performed using SigmaStat statistical software (version 2.0, Jandel Scientific, CA, USA). Results were represented as means ± standard deviation (SD). ANOVA was carried out when multiple comparisons were evaluated. Values were considered to be significant at *P* less than 0.05. All experiments were repeated at least three times independently.

## 3. Results

### 3.1. Cell Viability of A549 Cells Treated with TSL-1

**Figure 1:**
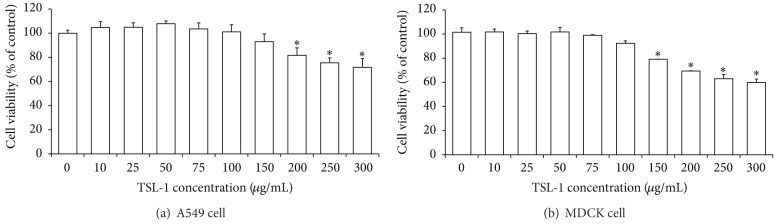
Effects of TSL-1 on the cell viability of A549 (a) and MDCK (b) cells. Cells treated with TSL-1 at different concentrations for 24 hours were determined by XTT cell proliferation assays. Data were the mean ± SEM calculated from three independent experiments in triplicate. Significance compared with vehicle control, *P < 0.05.

Regarding the cytotoxic effect of TSL-1, the water extract of TSL-1 did not show cytotoxicity against A549 and MDCK cells at concentrations up to 100 *μ*g/mL as shown in Figures 1(a) and 1(b). The phase-contrast microscopic examination (100X) on the cell morphology of A549 and MDCK cells after TSL-1 treatment did not reveal any obvious change, and there was no sign of apoptosis or necrosis (data not shown). The estimated CC50 of TSL-1 extracts was higher than 300 *μ*g/mL (Figure 1).

### 3.2. Inhibitory Effects of TSL-1 on Plaque Formation in MDCK Cells by Pandemic Influenza A (H1N1) Virus of Infected A549 Cells

**Figure 2:**
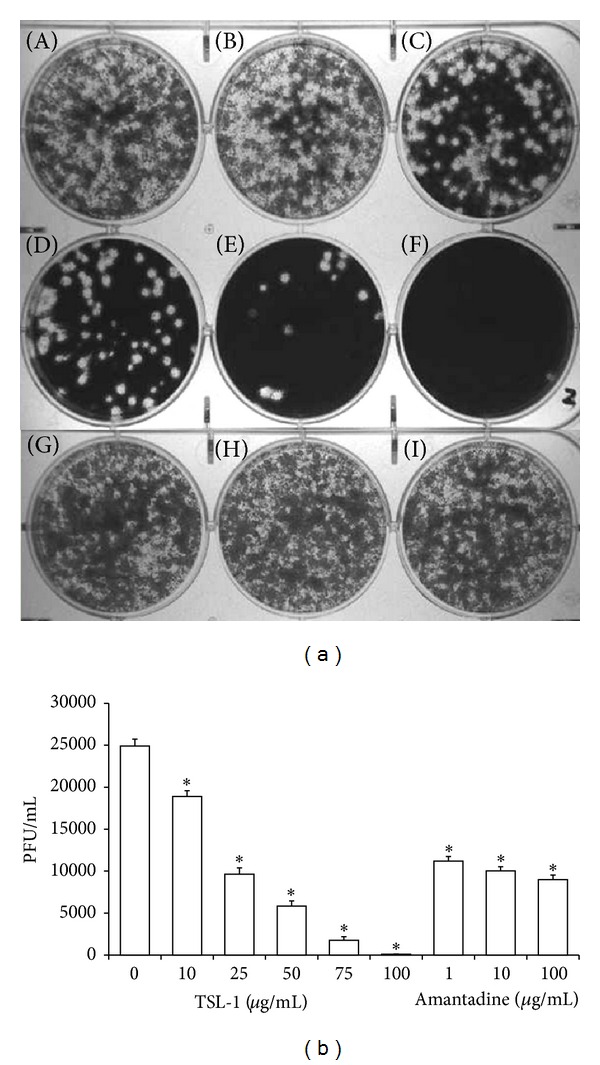
(a) TSL-1 inhibited pandemic influenza A (H1N1) virus replication. Plaque assays were performed on MDCK cells in the presence of various TSL-1 ((A) 0 *μ*g/mL, (B) 10 *μ*g/mL, (C) 25 *μ*g/mL, (D) 50 *μ*g/mL, (E) 75 *μ*g/mL, and (F) 100 *μ*g/mL) and Amantadine ((G) 1 *μ*g/mL, (H) 10 *μ*g/mL, and (I) 100 *μ*g/mL) concentrations. Viral yield was measured at 72 hours after infection. (b) Results were represented as PFU/mL with mean ± SEM from three independent experiments in triplicate. Significance compared with vehicle control, *P < 0.05.

To determine the effects of A/H1N1 viral growth of infected A549 cells by TSL-1, we first characterized viral plaque morphology in MDCK cells as shown in Figure 2. The change in viral yields on plaque formation demonstrated the inhibitory effect by TSL-1 with dose-dependent manner. The inhibitory effect of viral yields on plaque formation was demonstrated without dose-dependent manner. At the concentrations of 1, 10, and 100 *μ*g/mL, they displayed similar inhibitory effect in viral yields on plaque formation. Therefore, 10 *μ*g/mL of Amantadine through the whole research program was used as a positive control. (Figure 2(a)). We found that TSL-1 at the concentration of 25 *μ*g/mL decreased approximately 50% of the plaque formation of the viral yield. Virtually complete inhibition in viral yield was observed at the highest concentration of 100 *μ*g/mL with TSL-1 treatment (Figure 2(b)). The mean 50% inhibitory concentration (EC_50_) of TSL-1 is 20.4 ± 0.5 *μ*g/mL.

### 3.3. Selectivity Index against Pandemic Influenza A/H1N1 Virus of TSL-1

The selectivity index (SI) was calculated as the ratio of CC_50_ : EC_50_. The SI of TSL-1 was at least more than 15. It indicated that TSL-1 had strong inhibitory effect of pandemic influenza A (H1N1) virus without cytotoxic effect in MDCK cells.

### 3.4. Measurement of the Viral Genome Load in Infected A549 Cells with qRT-PCR

**Figure 3:**
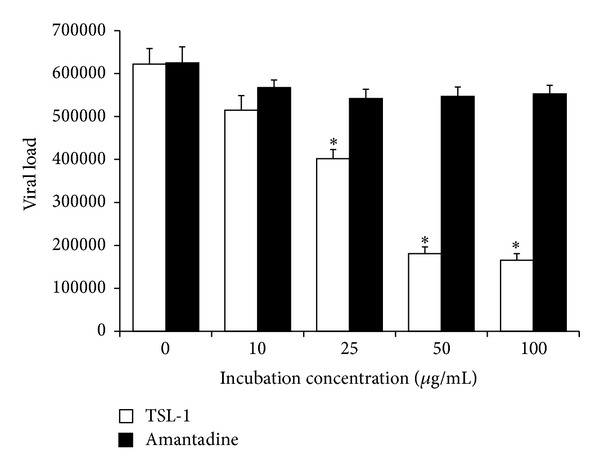
Inhibitory effect of TSL-1 on pandemic influenza A (H1N1) viruses RNA synthesis in A549 cells as analyzed by qRT-PCR. Data were the mean ± SEM calculated from three independent experiments in triplicate. Significance compared with vehicle control, *P < 0.05.

The TSL-1 effect on viral genome load in infected A549 was shown by log10 copy number decrements in treatments that were calculated through absolute quantification. A549 cells were infected with pandemic influenza A (H1N1) virus and incubated for 72 hours in the presence of various concentrations of TSL-1. Total RNAs were isolated from infected A549 cells, and qRT-PCR analysis was performed using specific primers for viral M gene RNA. The qRT-PCR results demonstrated significant inhibition of viral RNA synthesis in infected A549 cells at concentrations greater than 10 *μ*g/mL of TSL-1 treatment (Figure 3). However, there was no significant change of viral genome load in infected A549 cells with the 100 *μ*g/mL treatment of Amantadine.

### 3.5. TSL-1 Inhibited Pandemic Influenza A (H1N1) Virus through Attachment Not Penetration of A549 Cells

**Figure 4:**
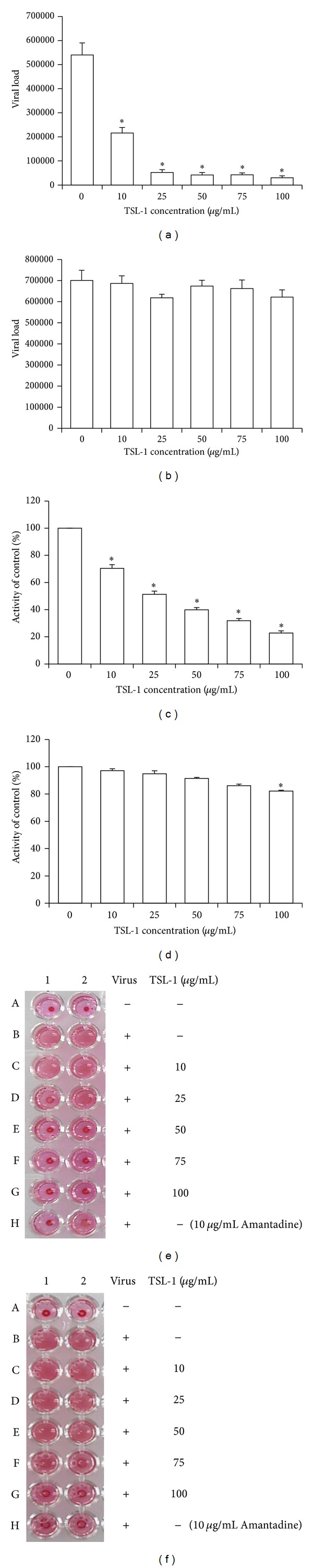
Effect of TSL-1 against pandemic influenza A (H1N1) infection was demonstrated by attachment assay and penetration assay. Effect of TSL-1 with the treatment procedures of attachment (a) or penetration (b) on influenza viral RNA synthesis in infected cells was analyzed by quantitative RT-PCR. Cell culture supernatants were collected and examined from the treatment procedures of attachment (c) or penetration (d) for virus yield by measured NA activity. Virus yield expressed as percent NA activity of culture supernatants of drug-free but only virus infected cells. Inhibitory effects of TSL-1 with the treatment procedures of attachment (e) or penetration (f) on virus adsorption to guinea pig red blood cells. Data were the mean ± SEM calculated from three independent experiments in triplicate. Significance compared with vehicle control, *P < 0.05.

According to the results of plaque assay and viral genome load, we demonstrated that TSL-1 has anti-influenza virus activity. We hypothesized that the TSL-1 might inhibit viral attachment and/or penetration of viral entrance into cells. As shown in Figure 4(a) with viral genome load detection by qRT-PCR, TSL-1 could significantly inhibit viral attachment to prevent the entrance of host cells with the treatment of less than 10 *μ*g/mL TSL-1. To the contrary, TSL-1 could not exert the effect of inhibiting viral penetration into A549 cells even up to 100 *μ*g/mL treatment of TSL-1 (Figure 4(b)). We further examined whether TSL-1 had any inhibitory activities towards NA through attachment or penetration process. Our results demonstrated that TSL-1 possessed inhibitory activities to NA through attachment effect with dose-dependent manner (Figure 4(c)) but not through penetration effect (Figure 4(d)). Influenza virus has an ability to adsorb to guinea pig red blood cells resulting in HA. We investigated if TSL-1 could interfere with the viral adsorption to RBC resulting in HA inhibition. The minimum concentration of 25 *μ*g/mL TSL-1 with the treatment procedure of attachment assay inhibits the adsorption ability of the virus (Figure 4(e)). The 100 *μ*g/mL TSL-1 with the treatment procedure of penetration assay resulted in the inhibition of adsorption of the virus (Figure 4(f)). We concluded from these results that TSL-1 inhibited pandemic influenza A (H1N1) virus activity through inhibiting attachment of A549 cells but not viral penetration.

### 3.6. Inhibitory Effect of Sialic Acid Activity and RANTES by TSL-1 and Amantadine on Pandemic Influenza A (H1N1) Virus

**Figure 5:**
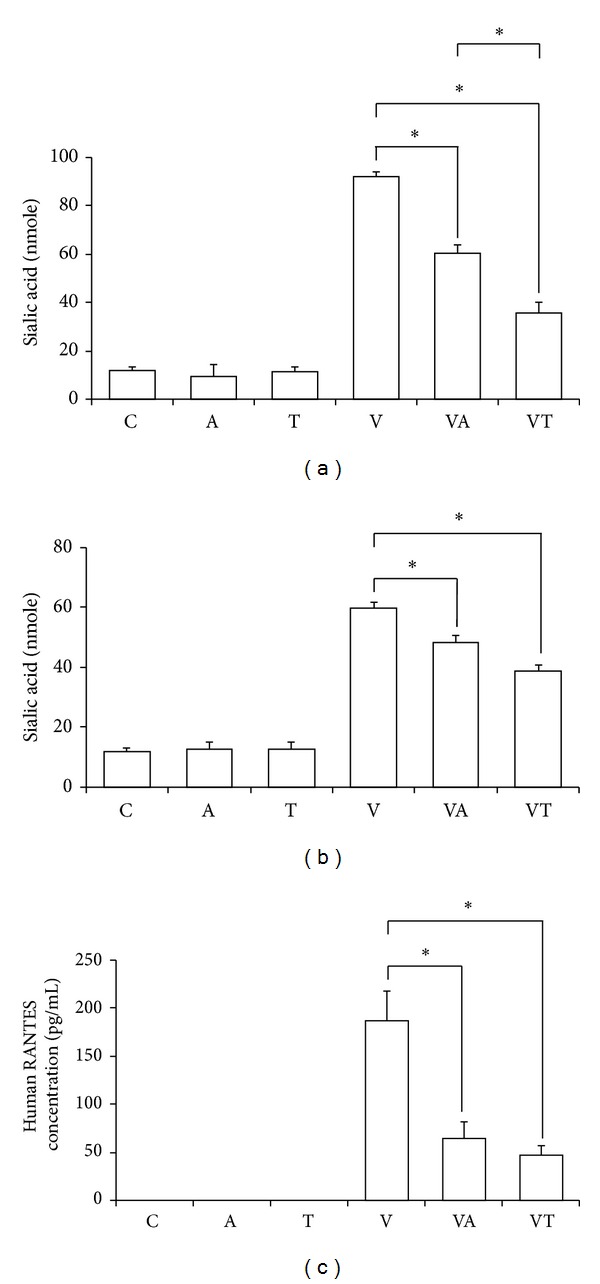
Inhibitory effect of sialic acid activity and RANTES by TSL-1 (25 *μ*g/mL) and Amantadine (10 *μ*g/mL) on pandemic influenza A (H1N1) viruses. Cell culture supernatants were collected and examined from the treatment procedures of attachment (a) or penetration (b) for virus yield to measure sialic acid activity. (c) RANTES concentration of supernatant in A549 cells by general viral infection of pandemic influenza A (H1N1) viruses was analyzed. Data were the mean ± SEM calculated from three independent experiments in duplicate. Significance compared with vehicle control, *P < 0.05.

The receptor hemagglutinin (HA) of influenza virus is sialic acid (SA). The initial attachment is the most important stage for virus infection of cells. Therefore, we tested whether SA could be suppressed by the treatment of viral infected cells with TSL-1. As shown in Figures 5(a) and 5(b), sialic acid was significantly suppressed by the treatment of TSL-1 in both viral attachment assay and viral penetration assay but more evidently in attachment assay. Sialic acid was also suppressed by treatment of Amantadine. However, its effectiveness is weaker than TSL-1. RANTES produced by virus-infected alveolar epithelial cells has been implicated in various pathophysiological processes including inflammation [22]. Furthermore, RANTES expression was suppressed in both TSL-1 and Amantadine treatments of viral infected A549 cells as shown in Figure 5(c). However, there is no statistical significance between TSL-1 and Amantadine.

### 3.7. mRNA Impacts of TSL-1 on A549 Cells with Pandemic Influenza A (H1N1) Virus Infection

**Figure 6:**
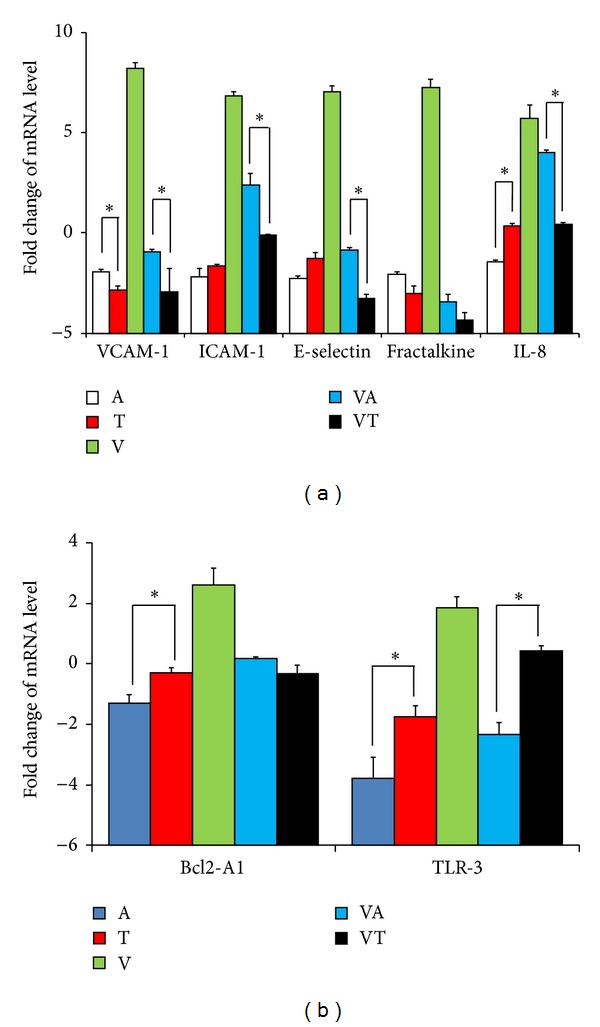
Effect of TSL-1 on the mRNA expressions of (a) VCAM-1, ICAM-1, fractalkine, E-selectin, IL-8, (b) Bcl2-A1, and TLR-3 in pandemic influenza A/H1N1-infected A549 cells. After culturing A549 cells with/without the treatment of 25 *μ*g/mL TSL-1 or 10 *μ*g/mL Amantadine in the presence or absence of H1N1 viral infection, the expression levels of mRNAs were measured by qRT-PCR with GAPDH normalization. Data were quantified and presented as mean values ± SEM of three independent experiments. Significance compared with vehicle control, *P < 0.05.

The process of inflammatory effect, immune activity, and host cell protection toward the respiratory endothelial cells in response to influenza A virus infection is associated with the release of proinflammatory cytokines and adhesion molecules along with a variety of chemokines [23, 24]. We found that mRNAs of ICAM-1, VCAM-1, E-selectin, fractalkine (CX3CL1), IL-8, TLR3, and Bcl2-A1 were activated by H1N1 viral infection and suppressed by TSL-1 treatment (Figure 6(a)). Amantadine has been reported as appropriate empiric treatment for influenza, associated infection of lower respiratory tract pneumonia [25]. Amantadine had more inhibitory effect on TLR3 gene expression of A549 cells in the presence and absence of H1N1 viral infection compared to TSL-1 (Figure 6(b)). The mRNA levels of ICAM-1, VCAM-1, E-selectin, fractalkine (CX3CL1), and IL-8 were inhibited by both TSL-1 and Amantadine. TSL-1 was more efficient to suppress the gene expression of these molecules in comparison to Amantadine (Figure 6(b)). As for the Bcl2-A1, we demonstrated that Amantadine was more effective to inhibit mRNA expression of Bcl2-A1 without H1N1 vial activation than TSL-1. However, TSL-1 was more efficient to reduce mRNA level of Bcl2-A1 with H1N1 vial activation compared to Amantadine (Figure 6(b)). This indicated that TSL-1 could have different mechanisms or pathways to prevent H1N1 viral infection other than Amantadine.

### 3.8. The Effects of TSL-1 on Protein Expression in the A549 Cells with or without Pandemic Influenza A (H1N1) Viral Infection

**Figure 7:**
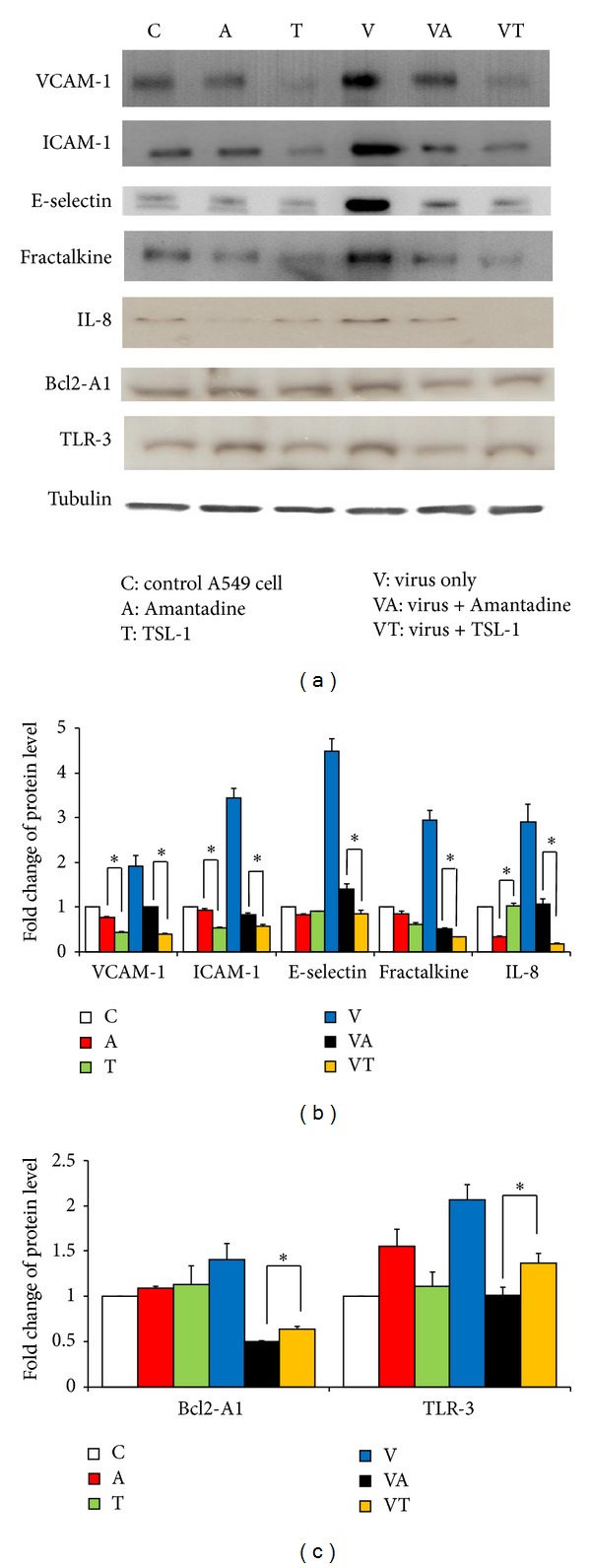
Effects of TSL-1 on the protein levels of VCAM-1, ICAM-1, E-selectin, fractalkine, IL-8, Bcl2-A1, and TLR-3 in pandemic influenza A (H1N1) virus-infected A549 cells. (a) After culturing A549 cells with/without the treatment of 25 *μ*g/mL TSL-1 or 10 *μ*g/mL Amantadine in the presence or absence of H1N1 viral infection, cell extracts were subjected to 12% SDS-PAGE and Western blot analysis with the respective primary antibody against VCAM-1, ICAM-1, E-selectin, fractalkine, IL-8, Bcl2-A1, and TLR-3. Tubulin protein was used as an internal control. Densitometric analyses of (b) VCAM-1, ICAM-1, E-selectin, fractalkine, IL-8, (c) Bcl2-A1, and TLR-3 were quantified and presented as mean values ± SEM of three similar independent experiments. Significance compared with vehicle control, *P < 0.05.

Based on the mRNA change inhibited by TSL-1 and Amantadine, we further explored the mechanism responsible for inhibition of adhesion molecules and chemokines expression. The Amantadine treatment of the unstimulated A549 cells did not show any difference in protein expression in VCAM-1, ICAM-1, and E-selectin in comparison to the control vehicle. However, TSL-1 was shown to be effective in suppressing the adhesion molecule secretions of unstimulated A549 cells. A large amount of VCAM-1, ICAM-1, E-selectin, IL-8, and fractalkine was secreted in the H1N1-stimulated A549 cells. Results show an inhibitory effect in expression of VCAM-1, ICAM-1, E selectin, IL-8, and fractalkine of the H1N1-stimulated A549 cells with the treatment of either Amantadine or TSL-1 (Figure 7). TSL-1 was more effective than Amantadine to inhibit adhesion molecules and chemokines in A549 cells with H1N1 viral activation (Figure 7) that is similar to the mRNA expression. We found that Amantadine inhibited the TLR3 and Bcl2-A1 expressions in the presence of H1N1 viral infection compared to the TSL-1. However, there is no specific inhibitory effect to the TLR3 and Bcl2-A1 expressions in the absence of H1N1 viral infection by the Amantadine and TSL-1.

## 4. Discussion

Outbreaks of pandemic influenza A (H1N1) virus pose a major public health risk. Infection with influenza A viruses is still a major health issue, and the treatment options are limited. Natural products and their derivatives have historically been important sources of therapeutic agents. The crude extract of *Toona sinensis Roem* leaf has been reported to exert antiproliferative action and growth inhibition on several cancer cell lines through apoptosis induction [9–13]. Recently, *Toona sinensis Roem* leaf extract was proven to abate hyperglycemia by altering adipose glucose transporter 4 [26] and improve lipolysis of differentiated 3T3-L1 adipocyte [27]. We also reported that TSL-1, the extract from tender leaf of *Toona sinensis Roem*, can inhibit SARS coronavirus [19], a virus that is known to cause influenza-like syndromes. Therefore, we researched whether TSL-1 could inhibit pandemic influenza A (H1N1) virus. To our knowledge, this is the first in vitro study that demonstrated anti-H1N1 activity with extracts from the tender leaf of *Toona sinensis Roem*.

Natural products and their derivatives have historically been invaluable as a source of therapeutic agents. As reported, a variety of polyphenols, flavonoids, saponins, glucosides, and alkaloids isolated from medicinal herbs have been studied extensively and shown to have anti-influenza activity. This is reflected by their ability to block adherence, penetration, duplication, and/or maturation during the course of viral propagation [28]. Epigallocatechin gallate (EGCG) is the ester of epigallocatechin and gallic acid that has been reported to inhibit the infectivity of both influenza A and B viruses in MDCK cells in vitro [29]. The derivatives of gallic acid, including lauryl gallate and Octyl gallate, have shown to significantly inhibit influenza virus production [30, 31]. These polyphenols as antioxidants protected human cells against oxidative damage might play a role to prevent influenza virus attack. Kaempferol, a flavonoid, exhibited the highest activity against two influenza viruses, H1N1 and H9N2 [32]. It was also found that 8-prenylkaempferol isolated from *Sophora flavescens* suppressed RANTES secretion in H1N1-infected A549 alveolar epithelial cells by blocking PI3K-mediated transcriptional activation of NF-*κ*B and IRF-3 (in part by interfering with I*κ*B degradation and subsequently decreases NF-*κ*B translocation) [22]. A recent study found quercetin and isoquercetin (quercetin-3-*β*-d-glucoside) significantly reduced the replication of influenza viruses in vitro and in vivo and protected the lung from the deleterious effects of oxygen-derived free radicals released during influenza virus infection [33, 34]. Additionally, rutin and quercetin exhibit a prooxidant effect in healthy animals and antioxidant activity in influenza-infected animals through detoxicosis increased hepatic monooxygenase activity [35]. TSL-1 includes the above compounds, as its major constituents. As for which specific compound inhibits H1N1 influenza virus, further investigation and identification are required.

In this study, we tested the viral inhibitory activity of TSL-1 in H1N1 viral infected MDCK cells. TSL-1 reduced CPE in MDCK cells and also reduced the viral replication in a dose-dependent manner with high selectivity index. This suggests that TSL-1 could inhibit viral adsorption to MDCK cells and thus block its infectivity. TSL-1 might have the effect in suppressing viral growth and protect cells from viral damage. To our knowledge, the early stages of influenza-host cell interaction include primary attachment of virus and subsequent irreversible attachment. We confirmed that TSL-1 blocked viral entry through attachment assay. TSL-1 may interact with viral proteins and alter the adsorption and penetration of the virion. In this study, we demonstrated that TSL-1 is more effective than Amantadine in inhibiting H1N1 infection. Moreover, the mechanisms of TSL-1 and Amantadine H1N1 inhibition are different. Our results demonstrated that TSL-1-mediated inhibition of H1N1 influenza virus-induced adhesion molecules and chemokines expressions was more prominent compared to Amantadine, and correlated well with decreased mRNA levels in A549 cells.

The adhesion molecules are upregulated on alveolar epithelial cells in response to TNF-*α* stimulation or during bacterial pneumonias in vitro and in vivo [22]. Increased adhesion molecules expression, such as ICAM-1 and VCAM-1, on A549 lung epithelial cells was reported upon respiratory syncytial virus infection [36]. Recently, ICAM-1 and VCAM-1 expressions were found to be upregulated on the surface of alveolar endothelial cells upon influenza A virus infection [22]. As we know, the major targets for influenza virus infection in host cells are located on the upper and lower airways. Influenza virus infection of epithelial cells and endothelial cells generally results in amplified viral replication and enhances the expression of adhesion molecules by the host cell to attract leukocytes for immunity. In our current study, analyses of cellular adhesion molecule expression on A549 in response to H1N1 virus infection revealed an upregulation of ICAM-1, VCAM-1, and E-selectin; this is subsequently inhibited by TSL-1. This indicates that TSL-1 is effective in decreasing the adhesion molecules expressed to protect the host from continual viral infection.

The process of inflammatory leukocyte recruitment toward the lungs in response to influenza A virus infection begins with the release of proinflammatory cytokines such as TNF-*α*, IL-6, IL-1, and along with a variety of chemokines like CCL2 (MCP-1), CCL5 (RANTES), CCL3/4 (MIP-1*αβ*), CXCL10 (IFN-inducible protein 10), and CXCL8 (IL-8) from infected alveolar macrophage and epithelial cells [22]. Beck et al. discussed the importance of chemokine biology in the progression of acute lung injury [37]. Recently, it was discovered that IL-8 and lung injury score independently predicted mortality in ARDS, and IL-8 was the most predictive of mortality [38]. Decreasing the release of TNF-*α* and IL-6 played a protective role to decrease vascular permeability and leukocyte recruitment through downregulation of fractalkine [39]. In our current study, IL-8 and fractalkine production after H1N1 infection was significantly downregulated by TSL-1. In brief, TSL-1 not only inhibits H1N1 viral infection but also attenuates the production of a critical chemokine, IL-8, and fractalkine that are responsible for acute lung injury. Therefore, TSL-1 might have the potential to not only decrease H1N1 infection but also to attenuate the severity of acute lung injury after H1N1 infection.

Expression of Toll-like receptor (TLR) family by endothelial cells plays a crucial role in the processes of inflammation through pathogen induction and activation of innate immunity. Bcl2-A1 is a member of the Bcl-2 protein family and acts as anti- and proapoptotic regulators that are involved in a wide variety of cellular activities, such as embryonic development [40], inflammation [41], and tumorigenesis [42]. This indicates that TSL-1's antiviral mechanism through modulating adhesion molecules and chemokines expressions is different to that of Amantadine, which modulates inflammation through TLR3 and Bcl2-A1.

In summary, we concluded that TSL-1 inhibited pandemic influenza A (H1N1) viral attachment and reduced cytokines responsible for acute lung injury. TSL-1 is more effective than Amantadine in inhibiting H1N1 via different pathways. The influenza virus infection imposes a considerable social and economic burden upon society. The nutritious vegetable *Toona sinensis Roem* may be an alternative supplement to traditional antiviral therapy.

## Figures and Tables

**Table 1:** Necleotide sequences of the primers and TaqMan probes.

Gene name	Sequence (5′-3′)	Accession no./nucleotide position	Amplicon size
VCAM-1	Forward: catgacctgttccagcgagg	NM_080682.2: 950–969	247 bp
	Reverse: cattcacgaggccaccactc	NM_080682.2: 1178–1197	

ICAM-1	Forward: ccttcctcaccgtgtactgg	J03132.1: 371–390	90 bp
	Reverse: agcgtagggtaaggttcttgc	J03132.1: 440–460	

E-selectin	Forward: ggttgagtgtgatgctgtga	NM_000450.2: 874–893	264 bp
	Reverse: gaaggtgaactctccagcag	NM_000450.2: 1119–1138	

Fractalkine (CX3CL1)	Forward: atcaacagaaccaggcatca	NM_002996.3: 219–238	151 bp
	Reverse: gccgccatttcgagttag	NM_002996.3: 353–370	

IL-8	Forward: agacagcagagcacacaagc	NM_000584.2: 4–43	62 bp
	Reverse: atggttccttccggtggt	NM_000584.2: 68–85	

TLR3	Forward: agttgtcatcgaatcaaattaaag	NM_003265.2: 707–732	62 bp
	Reverse: aatcttccaattgcgtgaaaa	NM_003265.2: 749–769	

Bcl-2-related protein A1	Forward: caggagaatggataaggcaaa	NM_001114735: 571–591	63 bp
	Reverse: accagcataggtgtgtgattgt	NM_001114735: 613–634	

5′-End labeled with fluorescein (FAM) and the 3′-end labeled with a dark quencher dye.
